# Host Preferences and Impact of Climate on Blood Feeding in *Anopheles funestus* Group from South Africa

**DOI:** 10.3390/tropicalmed9100251

**Published:** 2024-10-21

**Authors:** Tshiama Miriam Mwamba, Yael Dahan-Moss, Givemore Munhenga, Innocent Maposa, Lizette Leonie Koekemoer

**Affiliations:** 1 Wits Research Institute for Malaria, Faculty of Health Sciences, University of the Witwatersrand, 7 York Road, Parktown, Johannesburg 2193, South Africa; miriamm@nicd.ac.za (T.M.M.); yaeld@nicd.ac.za (Y.D.-M.); givemorem@nicd.ac.za (G.M.); 2 Division of the National Health Laboratory Service, Centre for Emerging Zoonotic and Parasitic Diseases, National Institute for Communicable Diseases, 1 Modderfontein Road, Sandringham 2192, South Africa; 3 Division of Epidemiology and Biostatistics, Department of Global Health, Faculty of Medicine and Health Sciences, Stellenbosch University, Stellenbosch 7599, South Africa; innocent.maposa@sun.ac.za; 4 Division of Epidemiology & Biostatistics, School of Public Health, Faculty of Health Sciences, University of the Witwatersrand, Johannesburg 2193, South Africa

**Keywords:** malaria, *An. parensis*, *An. rivulorum*, *An. vaneedeni*, *An. leesoni*, zoophilic

## Abstract

*Anopheles vaneedeni* and *Anopheles parensis* (members of the *An. funestus* group) are generally not considered malaria vectors. However, both species were recently identified as potential vectors in South Africa. A critical factor needed to determine their role in malaria transmission is their preference for human blood. The human blood index of *An. vaneedeni* and *An. parensis* and their potential role in the ongoing residual malaria transmission in South Africa is unknown. This study aimed to identify host blood meals from the wild-caught *An. funestus* group in a longitudinal study, and to establish the relationship between temperature, relative humidity, and precipitation on host feeding preferences. *Anopheles leesoni*, *An. parensis*, *An. vaneedeni*, and *Anopheles rivulorum* were collected, and females mainly fed on cattle. Climatic parameters did not influence the host feeding preferences of these four members of the *An. funestus* group, but impacted the proportion of females that took a blood meal. Significant changes in feeding proportions were driven by relative humidity, temperature, and precipitation. The role of these species in the ongoing residual malaria transmission in South Africa needs further investigation, as no human blood meals were identified. It is recommended that vector surveillance teams incorporate climatic monitoring and host blood meal identification into their routine activities. This information could provide the malaria vector control programmes with scientific evidence to evaluate the importance of the *An. funestus* group in residual malaria transmission.

## 1. Introduction

Malaria transmission in South Africa is low and mainly limited to the three endemic provinces of the country, namely Limpopo, Mpumalanga, and KwaZulu-Natal (KZN) [1]. Of the three provinces, KZN reports the lowest number of locally acquired malaria cases [2,3]. The ongoing residual malaria transmission is probably due to South Africa bordering malaria-endemic countries with high disease burdens (Zimbabwe, Eswatini, Mozambique, etc.), as well as outdoor transmission driven by vectors that bite and rest outdoors (which are not effectively killed by indoor control interventions) [3,4,5,6]. The situation is further complicated by potential secondary vectors that may also contribute to ongoing transmission in South Africa and neighboring countries. *Anopheles vaneedeni* and *An. parensis* are implicated as potential secondary vectors in South Africa [7,8].

To date, only two studies reported wild-caught *An. vaneedeni* and *An. parensis* infected with *P. falciparum* in South Africa [7,8]. Both studies used the head and thorax of females in a standard enzyme-linked immunosorbent assay (ELISA) method, PCR, and sequencing [7,8]. However, De Meillon and co-authors [9] showed that *An. vaneedeni* could experimentally be infected with malaria and, therefore, has the potential to be a vector. Since this species and others in the *An. funestus* group are mainly zoophilic (feeding mainly cattle), they are not considered to play an important role in malaria transmission [9,10]. In addition, *An. parensis* has also been found positive for *P. falciparum* in Tanzania [11,12]. This species was collected indoors in large numbers in South Africa and Kenya, placing it in close proximity to humans [13,14,15]. As with *An. vaneedeni*, host blood meal identification showed that *An. parensis* mainly fed on cattle blood, however, a few females tested positive for human blood meals (1.25%) [15]. Using the same ELISA technique as Mouatcho et al. [15], Kamau et al. [14] reported that 1.4% of the tested *An. parensis* were positive for human blood. Due to cross-hybridisation in ELISA assays, incorrect blood-meal identification is possible. Therefore, molecular PCR and sequencing approaches are currently used as confirmatory tests [16,17].

Other members of the *An. funestus* group, including *An. rivulorum* [11,18,19,20,21] and *An. leesoni* [11], are also implicated in malaria transmission. This raises important questions about their blood feeding preferences. Localised information on blood meal sources for members of the *An. funestus* group is somewhat limited, but necessary in terms of determining their role in malaria transmission, making blood meal identification an important vector surveillance indicator.

The infection and blood meal studies mentioned above, albeit limited, indicate that apart from *An. funestus*, other members of the *An. funestus* group are potential malaria vectors contributing to ongoing residual malaria transmission in South Africa. Reports of *An. vaneedeni* and *An. parensis* carrying *P. falciparum* infections in South Africa also raise the question of whether climatic factors, for example the period of drought experienced in South Africa from 2014 to 2019 [22,23,24], caused a change in host feeding preferences in populations of the *An. funestus* group from zoophily to anthropophily during this time [13,25]. This might explain the *P. falciparum* infections detected in *An. vaneedeni* and *An. parensis* reported by Burke et al. [7,8].

Mathematical models have been developed to explore the role of climate on malaria incidence and to aid in projecting future changes in malaria risk distribution and transmission. Seasonal malaria forecast models are being developed for South Africa using malaria early warning systems [26,27]. An important factor in these models is that peak malaria transmission in South Africa partly depends on the abundance of *Anopheles* vectors during the warmer and wetter summer season from November to April [28,29]. Climatic parameters affect the biology of mosquitoes, including their development and blood-feeding patterns [30,31]. Increased biting activity of *Anopheles* vectors typically occurs at warmer temperatures [6,31,32]. Warmer temperatures accelerate the gonotrophic cycle of *Anopheles* and other mosquitoes, thereby encouraging multiple blood feeding leading to a higher rate of disease transmission [31,33,34]. Temperature also affects vector resting behaviour, while the combination of temperature and relative humidity affects mosquito behaviour, survival, and proliferation [30,35]. Sufficient rainfall is required to form breeding sites for mosquitoes [30,36] and therefore affects their population abundance. These parameters are seldom collected routinely during entomological surveillance activities.

It was initially hypothesised that the drought experienced in South Africa in 2015 would result in subsistence farmers in the study area (northern KZN) relocating their farm animals, resulting in an increased probability of the resident zoophilic *Anopheles* species acquiring blood from humans instead, and therefore explaining the increase in *P. falciparum* infectivity in zoophilic species of the *An. funestus* group. Studies have indicated that drought indeed resulted in a loss of cattle and vegetation in KZN [22,24]. Furthermore, increased herd mortality because of a decrease in grass biomass was recorded mainly in 2016, which coincidentally falls within the period of this study [22,24]. As the samples used by Burke et al. [7,8] were not evaluated for blood meal source, their propensity to feed on humans, and therefore their contribution to residual malaria transmission, remains uncertain.

Implicating *An. vaneedeni* and *An. parensis* as potential secondary vectors in South Africa may affect the country’s malaria elimination strategy, especially if they are primarily outdoor-resting [7,8]. In addition, determining the blood meal sources of malaria vectors in a specific geographic region is an important facet of local malaria epidemiology, transmission dynamics, and vector control [37,38,39]. This study aimed to identify host blood meals from the wild-caught *An. funestus* group in a longitudinal study in northern KZN, and to establish the relationship between temperature, relative humidity, and precipitation on host feeding preferences.

## 2. Materials and Methods

### 2.1. Experimental Design, Study Site, and Sample Collection

**Figure 1:**
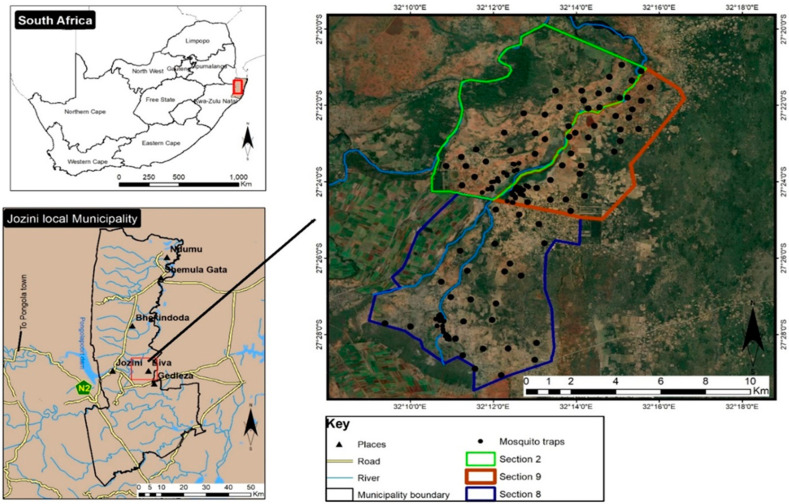
Map of Mamfene, northern KwaZulu-Natal Province, South Africa, showing three mosquito sampling sites: Sections 2, 8 and 9. The map was produced on Google Earth Pro (v7.3.3).

The mosquito samples used were obtained from archived storage, collected between 1 January 2015 and 31 December 2016 (called “archived samples” in this study), and female *An. funestus group* samples collected between May 2017 and June 2019, called “newly collected samples” in this study. Samples from January 2017 to 30 April 2017 were unavailable for this study, as they were used for the sterile insect technique (SIT) project. All the mosquito samples used in this study were collected from Mamfene, northern KwaZulu-Natal Province, South Africa (Figure 1). Specifically, the samples were collected from three sections in Mamfene (Sections 2, 8 and 9), which are being used as sentinel sites for entomological surveillance for the SIT study [6]. Adult *Anopheles* mosquitoes were collected from outdoor clay pots, carbon dioxide traps, outdoor buckets, and tyres as detailed by Dandalo et al. [6], and preserved for later analysis on desiccant (blue indicator silica gel).

### 2.2. Species Identification of the Anopheles funestus Group

While in the field, *An. funestus* group samples were morphologically identified using taxonomic keys [40,41] and transported to Johannesburg, South Africa for species identification. To identify the samples to species, DNA was extracted from each mosquito’s leg using the protocol detailed in the ZyGEM prepGEM^®^ Insect kit (Cat No.: PIN00200; ZyGEM™, Vienna, Austria). The extracted DNA was then used as a template in a PCR assay to differentiate between species [42]. Positive controls for the experiments were obtained from the FUMOZ colony, which was established using wild-caught *An. funestus* from Mozambique. *Anopheles leesoni*, *An. parensis*, *An. rivulorum*, and *An. vaneedeni* positive controls were obtained from samples identified from previous surveillance activities. Negative controls included the negative extraction control, where no specimen was added during DNA extraction, and a PCR negative control. The species identification of the archived samples was performed by Dandalo et al. [6] and Burke et al. [8], and the newly collected samples were identified as to species during this study.

### 2.3. Plasmodium Falciparum Sporozoite ELISA

*Anopheles funestus* group females identified as to species were analysed for *P. falciparum* circumsporozoite protein (CSP). *Plasmodium falciparum* results for the archived samples of the 2015 and 2016 collections were obtained from Burke et al. [8], whilst samples collected from May 2017 to June 2019 were processed during this study. To determine *P. falciparum* infectivity status, the heads and thoraces of all females were tested using the indirect sandwich ELISA method [43,44]. The antibodies were obtained through the Biodefense & Emerging Infections Research Resources Repository (BEI resources), National Institute of Allergy and Infectious Diseases, National Institute of Health (Atlanta, GA, USA): *Plasmodium falciparum* Sporozoite ELISA Reagent Kit, MRA-890, contributed by Robert A. Wirtz. All samples positive for *P. falciparum* were then processed using *P. falciparum*-specific nested PCR as described in [45].

### 2.4. Blood Meal Identification

#### 2.4.1. Sample Preparation

**Figure 2:**
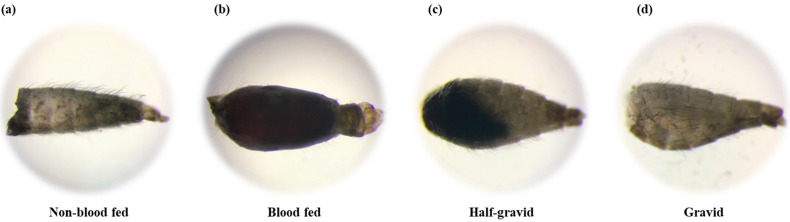
Blood-feeding status of *Anopheles* mosquitoes based on abdominal appearance under a dissecting microscope: (**a**) non-blood fed abdomen; (**b**) blood fed abdomen; (**c**) half-gravid abdomen; (**d**) gravid abdomen.

The blood digestion status of each female from the archived and newly collected samples was determined according to their abdominal appearances using a dissecting microscope. The status was classified as either non-fed (NF), fed (F), half-gravid (HG), or gravid (G) (Figure 2).

#### 2.4.2. DNA Extraction from Blood-Fed Females

Only fully blood-fed females were included in the analysis. Six positive controls were used for each test: Human, cattle, goat and dog blood-positive controls each originated from DNA extractions from *An. funestus* female specimens that had been positively identified to have fed on these hosts. Genomic DNA was extracted from the abdomen of the blood-fed females using Invitrogen’s PureLink™ Genomic DNA Mini Kit (Cat No: K1820-02, Thermo Scientific, Waltham, MA, USA). Engorged abdomens were dissected from the rest of the body and homogenised in phosphate-buffered saline (PBS, pH 7.2), and DNA was extracted according to the manufacturer’s protocol (Invitrogen’s PureLink™ Genomic DNA Mini Kit, Cat No: K1820-02, Thermo Scientific, Waltham, MA, USA).

Pig and chicken positive control: DNA was extracted from the blood of the purchased meat. A negative extraction control and PCR negative controls were also included. The samples were initially screened using human-, dog-, cow/cattle-, goat-, pig-, and chicken-specific primers according to Kent and Norris [16] and Cahyadi et al. [17] (Table 1). The PCR and cycling conditions used are described by Kent and Norris [16] and Cahyadi et al. [17], except that the annealing temperature was optimised for 57.5 °C for the Kent and Norris [16] assay.

### 2.5. Climate Data Sets

Meteorological data were collected for the period 1 January 2014 to 31 December 2019 from the automated HOBO weather station (Onset U30-NRC HOBO U30 USB Weather Station Data Logger) located in Section 8 (Figure 1) at the Malaria offices, in Mamfene, Jozini, KZN (S 27°27′34.3″; E 032°10′43.7″). The mean, minimum, and maximum temperatures, precipitation/rainfall, and humidity variables were extracted from the weather station data. Climate data for both the “archive” period (2015 and 2016) and the “new” period (2017–2019) were collected and filtered for missing values. A particular month was considered complete if there were three or fewer missing days per month (less than 10% of the month), and a full year was considered complete if only one month was missing for the period 2014–2019. All grid boxes met these conditions.

### 2.6. Data Analysis

All the statistical analyses were performed in STATISTICA version 13.0.17 and XLSTAT (Addinsoft, 23.3.1186.0, 2019). Statistically significant differences were set for 95% confidence. The Shapiro–Wilk test was used to test for normality. Blood meal analysis per collection method was not conducted because the clay pots (not so for the other traps) were intentionally placed in homesteads, which would have biased the analysis. The overall proportion of blood-fed females was calculated by dividing the proportion of blood-fed females by the total number of collected females. Mean monthly FPs were calculated as described in Equation (1), and mean annual FPs were obtained from the sum of monthly FPs divided by 12. Samples showing mixed blood meals were added to the totals of each of the corresponding individual vertebrate blood meals that were present in the mixed blood meals (refer to formula/equation 1 below). As no blood-fed females were collected in 2014 and between January and April 2017, these periods were excluded from the analysis. Mean monthly FPs were stratified into two collection periods representing the drought period “archived collection” (January 2015–December 2016, *n* = 24 months) and “new collections” (May 2017–April 2019, *n* = 24 months). Due to the low sample size, we combined the data into averages for the archived and new collection periods, resulting in 12 data points for each. For example, January 2015 and January 2016 were combined to obtain the average FP for January for the archived collection period. Changes in mean monthly FPs for each *Anopheles* species, irrespective of the vertebrate host they fed on, were determined for the archived versus the newly collected data using the Mann–Whitney U test.

The mean monthly FPs were expressed as a percentage for each species of the *An. funestus* group and were calculated as follows:

(1) $$\mathrm{FP}=\frac{N_{x}}{N_{T}},$$

where $N_{x}$ is the total number of a particular *Anopheles* species that were blood-fed (irrespective of host) per month; $N_{T}$ is the total number of all *Anopheles* species that were blood-fed per month.

Mean monthly FPs expressed as a percentage for each species of *An. funestus* group, per vertebrate host, was calculated as follows:

(2) $$\mathrm{FP}\;\mathrm{on}\;a\;\mathrm{specific}\;\mathrm{host}=\frac{N_{xh}}{N_{Tm}},$$

where $N_{xh}$ is the number of samples of *Anopheles* species that fed on a particular host per month; $N_{Tm}$ is the total number of blood-fed samples of that *Anopheles* species (per month).

Annual FPs are expressed as a percentage for each species of the *An. funestus* group, per vertebrate host and was calculated as follows:

(3) $$\mathrm{Annual}\;\mathrm{FP}\;\mathrm{on}\;a\;\mathrm{specific}\;\mathrm{host}=\frac{N_{xh}}{N_{Ty}},$$

where $N_{xh}$ is the number of samples of an *Anopheles* species that fed on a particular host per year; $N_{Ty}$ is the total number of blood-fed samples of the *Anopheles* species that year.

These were calculated for four collection years: year 1–2015 (January–December), year 2–2016 (January–December), Year 3 constituted samples from May 2017 and included samples until April 2018 for a 12-month collection period. The same was carried out for year 4: May 2018–April 2019.

The Chi-square test was used to determine if there was a difference in annual FPs per host for each *Anopheles* species across the four years.

For climatic data, temperature was summarised as mean monthly temperature (in °C), humidity as mean humidity (in %), and rainfall as monthly rainfall totals (in mm) calculated from the daily data. Weather seasons were defined based on meteorological divisions: Summer = 1 December–28/29 February; Autumn = 1 March–31 May; Winter = 1 June–31 August; and Spring = 1 September–30 November. However, it should be noted that the seasons are not strictly delimited to those periods. Changes in climatic parameters were evaluated to determine which periods in Mamfene experienced drought, defined as the occurrence of less-than-average rainfall, i.e., less than 476.8 mm/year for Mamfene [23]. Drought is mainly characterised by increased atmospheric temperatures and low relative humidity. The Mann-Kendall trend test was applied to investigate if annual temperatures, relative humidity, and total rainfall changed over the study period. ANOVA and Tukey’s HSD (honestly significant difference) test were performed to analyse the trends between climatic parameters and blood meal host preference across study years, assuming a normal distribution of climatic parameters. Friedman’s test for non-parametric climatic parameter(s) was performed. Spearman’s correlation was used to determine the association between FPs of each species of the *An. funestus* group and the climatic parameters in the archived and newly collected data. Time-series analysis was then employed to depict significant correlations where they existed.

### 2.7. Ethical Clearance

Ethical clearance for this study was waived by the animal research ethics committee at the University of the Witwatersrand (AREC-101210-002).

## 3. Results

### 3.1. Species Composition and P. falciparum Infectivity Analysis

A total of 759 female mosquitoes belonging to four species of the *An. funestus* group were collected during both collection periods in Mamfene. Most of the samples (75.2%, 571/759) were sampled during the “new collection” period (Table 2). *Anopheles parensis* was the most prevalent species in both the archived and new collections, 34.0% (64/188) and 63.0% (360/571), respectively (Table 2). The other three *An. funestus* group members were *An. leesoni*, *An. rivulorum*, and *An. vaneedeni*. Amongst the newly collected samples, two *An. rivulorum* (collected in 2018 and 2019) and one *An. parensis* from 2019 tested positive for *P. falciparum* CSP, even after repeating the ELISA test with a heating step to eliminate false positives [44]. However, all three samples tested negative using the nested PCR assay confirmation test [45].

### 3.2. Host Preferences of Species of the An. funestus Group in Mamfene

The abdominal status (blood-fed, unfed, gravid, or half-gravid) of the 759 females in this study was recorded. Of these, 37.9% were non-fed (288/759), 32.8% blood-fed (249/759), 21.3% gravid (162/759), and 7.9% half-gravid (60/759) (Table 3). The blood meal source was successfully identified in 87.6% (218/249) of the blood-fed samples belonging to all four *Anopheles funestus* group species identified in this study, whilst only 12.4% (31/249) had unidentifiable blood meal origins.

Out of all the specimens, no mosquitoes were identified as blood-fed on either human or chicken blood. The most common vertebrate host identified in all four species of the *An. funestus* group was cow, followed by goat (Table 3). Subsequent analysis was conducted only on samples where PCR successfully identified the blood meal source. Most of the female *An. leesoni* had fed on cattle (90.9%, 20/22), whilst the remaining fed on goat (9.1%, 2/22). Cattle were also the primary host for *An. parensis* (77.5%, 93/120) followed by goat (11.7%, 14/120), pig (1.7%, 2/120) and one that fed on dog blood. Eighty-six per cent (32/37) of *An. rivulorum* had fed on cattle, and the majority of *An. vaneedeni* also fed on cattle (85%, 33/39). These species, except for *An. leesoni*, had blood-fed on mixed-blood meals in some samples. Multiple-host blood was also detected in 13 females (6.0%, 13/218) (Table 3, Supplementary Table S1). The most common blood meal combination, comprising cow and goat blood, was recorded for *An. parensis* (69.2%, 9/13) and *An. rivulorum* (7.7%, 1/13). Two samples (*An. rivulorum* and *An. vaneendeni*) had a mixed-blood meal of cow and pig, while one *An. parensis* sample had a mixed meal consisting of pig and dog blood.

### 3.3. Temporal Pattern and Change in Climatic Parameters

**Figure 3:**
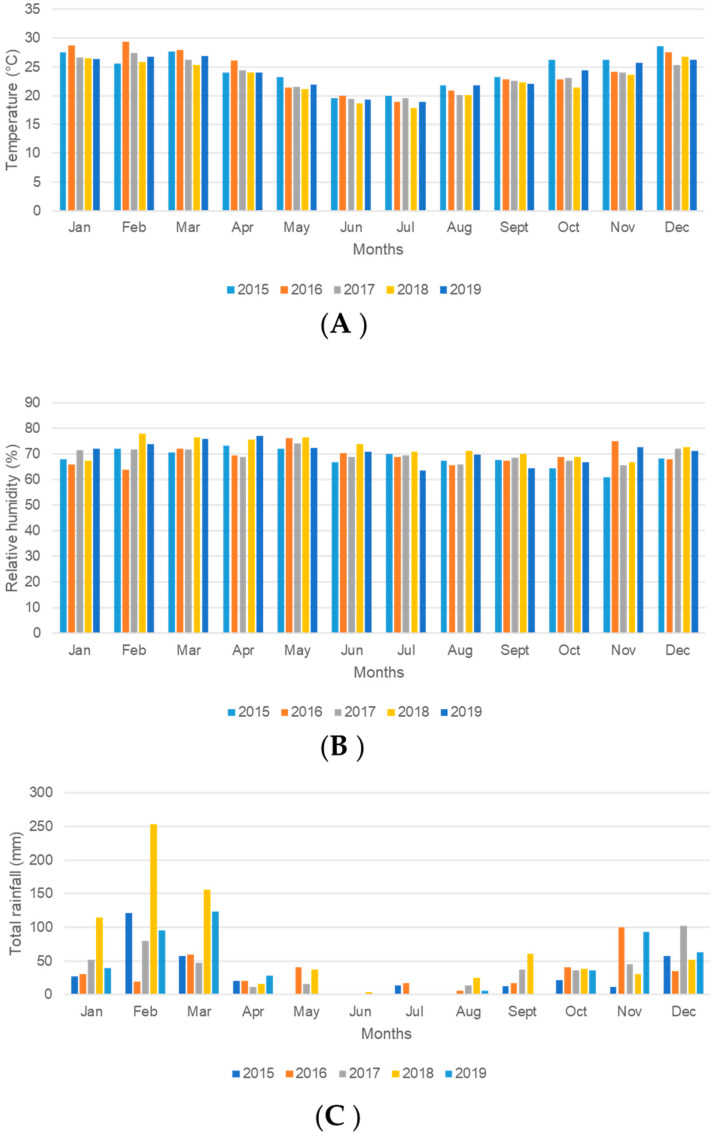
Temporal trend of three climatic parameters by month and year in Mamfene, KwaZulu-Natal Province, South Africa. Average monthly values for January 2015–December 2019. (**A**) Temperature (°C), (**B**) Relative humidity (%), (**C**) Total monthly rainfall (mm).

The highest temperatures, peaking at 28.8 °C, occurred in summer (December–February), whilst the lowest temperatures were in winter (June–July) at 17.4 °C (Figure 3A). A one-way ANOVA revealed that there was a statistical difference in mean monthly temperatures between the years (F_(5, 55)_ = [9.982], *p* < 0.001). Pairwise comparisons of mean monthly temperatures for 2015–2019 using Tukey’s HSD test revealed that the mean monthly temperatures were statistically different between three pairs of years (Supplementary Table S2). The years 2015 and 2016 were paired in the same group (archive period) and experienced the highest mean monthly temperatures compared to the other years (*p* < 0.01, 95% CI) (Figure 3A).

Relative humidity (%) was higher during March–May (Autumn), peaking at 79.3% compared to other seasons. Mean monthly relative humidity values decreased in winter (June–August) and into spring (September–November) (Figure 3B). There was a statistical difference in mean monthly relative humidity values between the years (F_(5, 55)_ = [2.893], *p* = 0.0213) based on a one-way ANOVA. Tukey’s HSD test for multiple group comparisons found that the mean relative humidity values were statistically different only between 2015 and 2018 (*p* = 0.017, 95% CI) (Supplementary Table S3), with 2015 having the lowest mean relative humidity compared to 2018 (Figure 3B).

Very little to no rainfall occurred in June, whilst high rainfall was primarily recorded in summer (December–February) and the beginning of autumn (March) (Figure 3C). The year 2015 experienced the lowest total annual rainfall (343.4 mm) compared to the other years, and the highest annual total rainfall (485.4 mm) was experienced in 2019. However, Friedman’s test showed no statistical difference in mean annual rainfall between the different years (Chi Sqr. Χ^2^ (12, 5) = 9.196, *p* = 0.102).

### 3.4. Influence of Climatic Parameters on Annual FPs of Species of the An. funestus Group, per Host Preference

The influence of climatic parameters on annual FPs for each blood-meal source for each member of the *An. funestus* group (Table 4) was also investigated. In contrast to Table 3, the mixed-blood meals in Table 4 were reallocated to individual blood meal sources to prevent underestimating the proportion for each blood meal source.

Overall, there was no change in annual FPs for all *An. funestus* group species on cattle, goat, dog or pig, except for *An. parensis* that fed on goat.

#### 3.4.1. Yearly Cattle Blood-Feeding Dynamics

There was no statistically significant difference in yearly cattle FPs in all four species based on Chi-square analysis. Although there were no annual changes in the cattle FP for *An. vaneedeni*, it showed a strong positive association with mean annual temperature (Pearson’s correlation, r = 0.986, *p* < 0.05) and a strong negative correlation with mean annual relative humidity (Pearson’s correlation, r = −0.958, *p* < 0.05) (Supplementary Figure S1).

#### 3.4.2. Yearly Goat Blood-Feeding Dynamics

The annual FPs of *An. parensis* on the different hosts did not change over the years under investigation. However, the exception was its FPs on goats, which was significantly higher in year 4 (May 2018–April 2019) compared to year 3 (May 2017–April 2018), ($X^{2}$, df = 9, *p* = 0.044). However, no association was observed between FPs and any of the climatic parameters.

#### 3.4.3. Influence of Climatic Parameters on Monthly Feeding Proportions in Archived and Newly Collected Data, per Anopheles Species, Irrespective of Host

- *Anopheles parensis*

**Figure 4:**
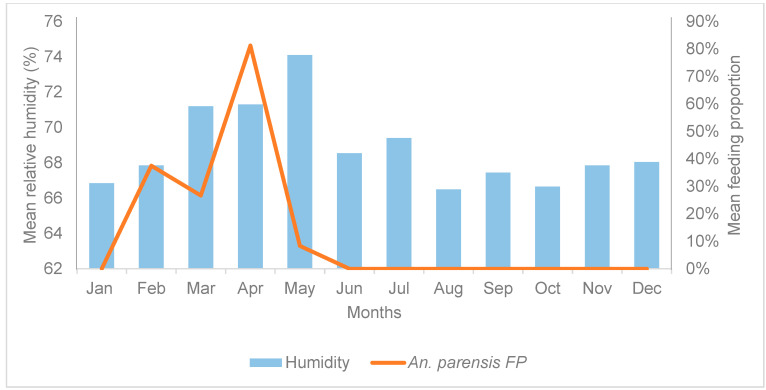
Time series plot for mean monthly FPs of *Anopheles parensis* and mean monthly relative humidity in Mamfene, KwaZulu-Natal Province, South Africa, in the archived collection (January 2015–December 2016). FPs: feeding proportion expressed as percentages.

The mean FPs for *An. parensis* were significantly lower for the archived period (Jan 2015–Dec 2016) compared to the new period (May 2017–April 2019) (Mann–Whitney test, *U* = 14.000, *Z* = −3.320, *p* = 0.001). Spearman’s correlation revealed a positive correlation between the mean FPs of *An. parensis* and mean relative humidity in the archived data (r = 0.593, *p* < 0.05, 95% CI) (Figure 4, Supplementary Table S4).

- *Anopheles vaneedeni*

**Figure 5:**
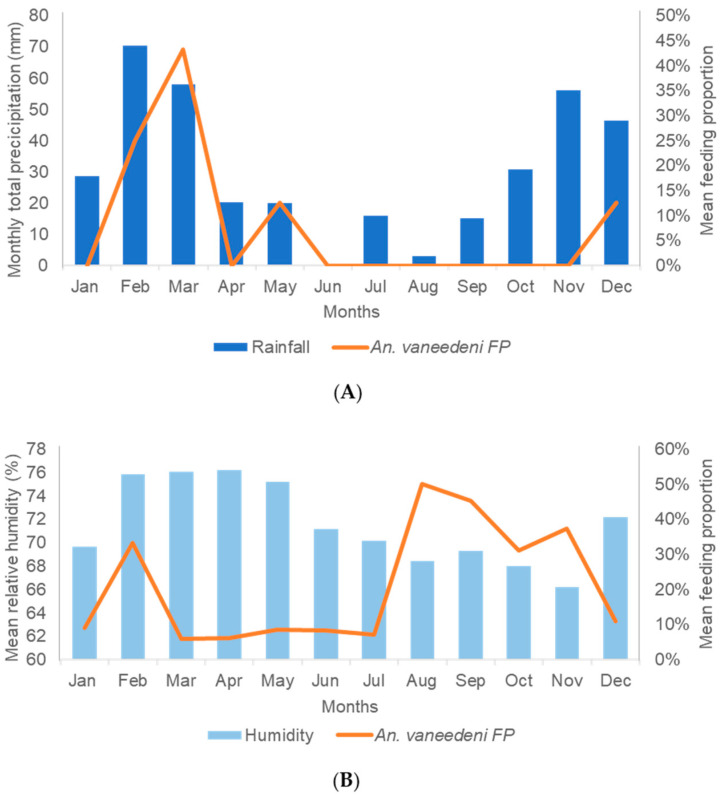
Mean monthly FPs of *Anopheles vaneedeni* and climatic parameters in Mamfene, KwaZulu-Natal Province, South Africa. (**A**) Mean monthly FPs of *An. vaneedeni* and mean monthly rainfall in the archived period (January 2015–December 2016). (**B**) Mean monthly FPs of *An. vaneedeni* and mean monthly relative humidity in the new period (May 2017–April 2019). FP = feeding proportion.

The mean FPs for *An. vaneedeni* were significantly lower in the archived period (January 2015–December 2016) when compared to those in the new sampling period (May 2017–April 2019) as per the Mann–Whitney U test (*U* = 31.000, *Z* = −2.338, *p* = 0.019). The lower FP of *An. vaneedeni* in the archived data was correlated with mean total rainfall, with a strong positive correlation (r = 0.621, *p* < 0.05, 95% CI) (Figure 5A, Supplementary Table S3). Increasing rainfall of up to 70 mm was favourable for *An. vaneedeni* feeding up to 45% FP (Figure 5A). The FPs of *An. vaneedeni* showed a strong negative correlation with relative humidity in the new collection period (Pearson correlation, r = −0.692, *p* < 0.05, 95% CI) (Figure 5B, Supplementary Table S5).

- *Anopheles rivulorum*

**Figure 6:**
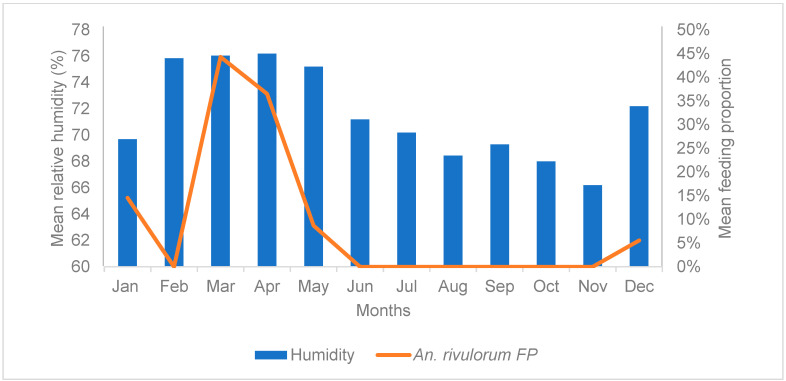
Mean monthly FPs of *Anopheles rivulorum* and mean relative humidity in newly collected data (combined May 2017–April 2019) from FPs, Mamfene, KwaZulu-Natal Province, South Africa. FP = feeding proportion.

The mean FPs for *An. rivulorum* did not statistically differ between the archived and the new period (Mann–Whitney U test, *U* = 67.000, *Z* = −0.260, *p* = 0.765). However, FPs of *An. rivulorum* showed a strong positive correlation with mean monthly relative humidity in the new period (Pearson correlation, r = 0.655, *p* < 0.05, 95% CI) (Figure 6, Supplementary Table S5). The highest FPs were recorded during autumn, 44% in March and 37% in April, corresponding with the highest relative humidity values, >76% for both months (Figure 6).

- *Anopheles leesoni*

The mean monthly FPs of *An. leesoni* was higher in the archived period than in the new period, but this difference was not statistically different (Mann–Whitney U test, *U* = 40, *Z* = 1.891, *p* = 0.059).

## 4. Discussion

In South Africa’s malaria elimination setting, it is necessary to establish the role of primarily zoophilic species in malaria transmission. From samples of the four species of the *An. funestus* group collected outdoors in Mamfene, northern KZN, South Africa (*An. parensis*, *An. vaneedeni*, *An. rivulorum*, and *An. leesoni)*, none were infected with *P. falciparum* sporozoites according to the PCR assay. However, three samples were positive for *P. falciparum* using ELISA, highlighting the necessity of using molecular confirmation post-CSP-ELISA for unexpected positives (i.e., those species not normally implicated in malaria transmission). This necessity is reinforced by the blood meal source analysis showing that none of the females tested had fed on humans. Most had primarily fed on cattle, goats, pigs, and dogs.

During the study period, the primary malaria vector *An. funestus sensu stricto* was not detected. *Anopheles funestus* is generally considered near-eradicated within South Africa. The last report of this species was in 2018 when one *An. funestus* was collected from Limpopo Province [46], and its occurrence was attributed to the collection site that is in close proximity to the Zimbabwe border where this species commonly occurs [47]. Even in the absence of *An. funestus s.s.*, other species of the *An. funestus* group in Mamfene are routinely surveyed to monitor their potential role in malaria transmission [37,48]. The discrepancy between *P. falciparum*-positive ELISA samples that were negative on PCR in this study might be due to the methods used to extract DNA from ELISA homogenates for molecular confirmation (Aswat et al., unpublished). Therefore, their incrimination as a secondary malaria vector in South Africa remains uncertain, and their role in the ongoing residual malaria transmission in the KZN province has not been fully substantiated. Nonetheless, it would be beneficial to closely monitor the vectorial capacity and host preferences of all the outdoor-feeding and resting species of the *An. funestus* group. In addition, future studies can include collection methods designed to collect human-feeding mosquitoes (e.g., human landing catches or proxy methods) to better understand how various members of the *An. funestus* group interact with humans. Salivary gland dissection of wild-caught females can provide further information on the role of these species in malaria transmission.

Regardless of species, all four members of the *An. funestus* group identified showed a strong preference for feeding on cattle, followed by goats. These findings confirm a previous study conducted in Limpopo Province [10] and constitute the first report that is based on the use of molecular methods to show that *An. vaneedeni* from KwaZulu-Natal mainly feeds on cattle. However, the results from this study should be interpreted with caution. The sampling methods for mosquito collection were limited to outdoor resting traps, because indoor collections in South Africa are not productive owing to indoor residual spraying for vector control. Nevertheless, in other studies, one *An. vaneendeni* sample from Malawi (0.2% of the total sample size) was shown to have fed on humans [38] and a more recent study in Angola identified two indoor-collected *An. vaneedeni* specimens that had fed on human and bovine blood [39]. Host preference studies for this species, and others in the *An. funestus* group, are currently limited and should be expanded to include additional African localities.

Additionally, to the best of our knowledge, there is no published information on the host preference of *An. leesoni* and *An. rivulorum* in South Africa. There is generally limited information on the preferred blood meal source by *An. rivulorum* despite this species being consistently implicated as a secondary vector in various African countries [11,18,19,20,21]. *Anopheles leesoni* collected from Ethiopia showed that 13% of specimens had fed on human blood [49], and 43.5% had fed on bovine blood. A small number of *An. rivulorum* from Kenya fed on humans, while the remaining samples (87%) fed on cattle or other animals [20]. Previous studies from the same study site confirm that *Anopheles parensis* feeds predominantly on cattle [15], and this was confirmed in the present study. A study from Kenya showed that 98.5% of *An. parensis* collected indoors fed on animals [14], and this was supported by a study from Malawi, which showed a similar result [50]. However, this contradicts Mbewe et al. [38], who found one sample in Ethiopia that had fed on a human host.

Interestingly, despite an abundance of chickens in households in Mamfene (Munhenga *pers. comm*), none of the collected females had blood-fed on chickens. This contradicts a previous study [15] where it was observed that 3.75% of *An. parensis* females had fed on chickens (in the same locality) using blood meal ELISA assays. The lack of *An. funestus* group females taking their blood meals from chickens could be explained by volatiles produced by chickens known to repel mosquitoes [51]. Chicken volatiles repel host-seeking *An. arabiensis* and may have a similar repellent effect on species of the *An. funestus* group. Further investigations are needed to confirm this hypothesis.

Climatic factors play an important role in malaria epidemics [30,35], and the role of these factors in host preference and blood feeding was investigated. No statistical difference in monthly rainfall was observed between the period 2015 to 2019 and the period 2015 to 2016. However, the total annual rainfall (343.4 mm and 386.6 mm, respectively) was below Mamfene’s usual average annual rainfall (476.8 mm/year). Moreover, 2015 had the lowest relative humidity compared to subsequent years of the study. These dry conditions confirm published records that the 2015 and 2016 rains were the lowest in the past 48 years in KZN (1970–2017) [23]. These rainfall figures were also below the South African and world annual averages of 500 mm/year and 860 mm/year, respectively [23]. Apart from low rainfall, this study recorded the highest mean monthly temperatures in KZN during 2015 and 2016.

These climatic parameters influenced the blood feeding of members of the *An. funestus* group differently. Relative humidity and rainfall were the primary factors and were associated with changes in blood-FPs. The effect of each climatic factor was species-dependent. For example, the mean monthly FPs for *An. vaneedeni* were negatively correlated with relative humidity, whereby high humidity resulted in lower blood-feeding propensity. Conversely, *An. parensis and An. rivulorum* FPs were positively correlated with relative humidity. More blood-fed females of these two species were recorded during periods of high relative humidity, and lower FPs were observed during drought conditions, i.e., low humidity. These findings are supported by the literature, where relatively high humidity is a favourable condition that allows mosquitoes to survive longer, thus reproducing more [36,52], and consequently increasing the need for frequent blood meals to develop more eggs. Studies have shown that a relative humidity of 60–70% is favourable for mosquitoes’ survival, giving them prolonged time to feed more frequently [30,36,52].

Furthermore, the results showed that only the annual FP of *An. vaneedeni* that fed on cattle correlated with changes in temperature and humidity. There appeared to be an association between higher temperatures (~23 °C) and lower humidity (<70%) with increased feeding on cattle for *An. vaneedeni*. This correlates with other studies that showed that mosquito biting rates and feeding frequencies generally increase with rising temperatures [32,53]. The warmer temperatures (>25 °C) increase cell metabolism, causing faster digestion of blood in mosquitoes, resulting in an increase in the frequency of feeding [53]. However, the specific behavioural changes depend on each species’ thermal limit [54]. Unfortunately, the thermal limits of the zoophilic members of the *An. funestus* group have not yet been recorded, making it difficult to draw conclusions. Another limitation of this study is the size of the samples available for this study, which highlights the importance of collecting all anophelines during vector control surveillance activities.

## 5. Conclusions

The study confirmed the occurrence of four species of the *An. funestus* group in KZN. These species largely remain zoophilic, feeding primarily on cattle, suggesting that their role in ongoing residual malaria transmission in KZN might be minimal. Blood feeding propensity by females of these species is influenced by specific climatic factors differently, with relative humidity and rainfall being the primary drivers. The relationship between climatic factors and vertebrate host preference should be incorporated into malaria risk models. This study emphasises the importance of monitoring climatic factors and mosquito blood meal sources to better understand malaria transmission dynamics, especially in low-incidence settings where control operations are in place.

## Figures and Tables

**Table 1:** Primer sequences by blood source and their target genes for use in conventional *Anopheles* blood meal PCR assays, with amplicon sizes and respective melting temperatures (Tm) listed.

Blood Source	Primer Name	Oligonucleotide Sequence (5′-3′)	Target Gene	Tm (°C)	Amplicon Size (bp)
Human	Human741F	GGCTTACTTCTCTTCATTCTCTCCT	cyt b^16^	64.2	334
Dog	Dog368F	GGAATTGTACTATTATTCGCAACCAT	cyt b^16^	61.6	680
Cow	Cow121F	CATCGGCACAAATTTAGTCG	cyt b^16^	56.4	561
Goat	Goat894F	CCTAATCTTAGTACTTGTACCCTTCCTC	cyt b^16^	67.2	132
Pig	Pig573F	CCTCGCAGCCGTACATCTC	cyt b^16^	61.7	453
	UNREV1025	GGTTGTCCTCCAATTCATGTTA	cyt b^16^	59.4	-
	UNFOR	ACCGCGGTCATACGATTAAC	12S rRNA^17^		-
Cow	SP_R	AGTGCGTCGGCTATTGTAGG	12S rRNA^17^		155
Pig	BB_R	GAATTGGCAAGGGTTGGTAA	12S rRNA^17^		357
Chicken	A_R	CGGTATGTACGTGCCTCAGA	12S rRNA^17^		611

**Table 2:** Samples of members of the *Anopheles funestus* group by species and collection period, Mamfene, KwaZulu-Natal Province, South Africa.

Species	Archived Collection (January 2015–December 2016)	New Collection (May 2017–April 2019)	Total
*An. leesoni*	41	24	65
*An. parensis*	64	360	424
*An. rivulorum*	39	98	137
*An. vaneedeni*	44	89	133
Total	188	571	759

**Table 3:** Abdominal status by *Anopheles funestus* group species and corresponding sources of vertebrate blood based on PCR analysis.

		Abdominal Status, *n*				Blood Meal (BM) PCR, *n* (%)							
Species	Non-Fed	Blood-Fed	Gravid	Half-Gravid	Total	Dog	Cow	Goat	Pig	Mixed	BM Identified (%)	BM Not Identified (%)	Total
*An. leesoni*	21	24	16	4	65	0 (0)	20 (90.9)	2 (9.1)	0 (0)	0 (0)	22 (91.7)	2 (8.3)	24
*An. parensis*	161	135	90	38	424	1 (0.8)	93 (77.5)	14 (11.7)	2 (1.7)	10 (8.3)	120 (88.9)	15 (11.1)	135
*An. rivulorum*	60	43	25	9	137	0 (0)	32 (86.5)	2 (5.4)	1 (2.7)	2 (5.4)	37 (86.0)	6 (14.0)	43
*An. vaneedeni*	46	47	31	9	133	0 (0)	33 (84.6)	5 (12.8)	0 (0)	1 (2.6)	39 (83.0)	8 (17.0)	47
Total	288	249	162	60	759	1 (0.5)	178 (81.7)	23 (10.6)	3 (1.4)	13 (6.0)	218 (87.6)	31 (12.4)	249

BM: Blood meal.

**Table 4:** Average annual proportions of host feeding by species of the *Anopheles funestus* group (*n*/*N*, % rounded off to the nearest percentage) by vertebrate host, with corresponding annual climatic parameters, Mamfene, KwaZulu-Natal Province, South Africa.

Host	Year	*An. Leesoni*	*An. Parensis*	*An. Rivulorum*	*An. Vaneedeni*	Annual Mean Temperature (°C)	Annual Mean Relative Humidity (%)	Annual Total Rainfall (mm)
Cattle	Year 1–2015	11/13 (85%)	8/11 (73%)	2/3 (67%)	9/9 (100%)	24.5	68.4	343.4
	Year 2–2016	0	2/3 (67%)	3/3 (100%)	2/2 (100%)	24.3	69.2	386.6
	Year 3–May 2017–April 2018	5/5 (100%)	52/58 (90%)	15/16 (94%)	5/6 (83%)	23.1	70.7	792.8
	Year 4–May 2018–April 2019	4/4 (100%)	40/58 (69%)	14/17 (82%)	18/23 (78%)	23.0	72.4	534.8
Goat	Year 1–2015	2/13 (15%)	3/11 (27%)	1/3 (33%)	0	24.5	68.4	343.4
	Year 2–2016	0	1/3 (33%)	0	0	24.3	69.2	386.6
	Year 3 May 2017–April 2018	0	5/58 (9%)	0	1/6 (17%)	23.1	70.7	792.8
	Year 4 May 2018–April 2019	0	14/58 (24%)	2/17 (12%)	4/23 (17%)	23.0	72.4	534.8
Dog	Year 1–2015	0	0	0	0	24.5	68.4	343.4
	Year 2–2016	0	0	0	0	24.3	69.2	386.6
	Year 3 May 2017–April 2018	0	1/58 (2%)	0	0	23.1	70.7	792.8
	Year 4 May 2018–April 2019	0	1/58 (2%)	0	0	23.0	72.4	534.8
Pig	Year 1–2015	0	0	0	0	24.5	68.4	343.4
	Year 2–2016	0	0	0	0	24.3	69.2	386.6
	Year 3 May 2017–April 2018	0	0	1/6 (6%)	0	23.1	70.7	792.8
	Year 4 May 2018–April 2019	0	3/58 (5%)	1/17 (6%)	1/23 (4%)	23.0	72.4	534.8
Total		22	130	39	40			

Note: Mixed-blood meal data from Table 3 were reallocated to represent the host blood meal per species. Thus, the numbers in the table represent the “total number of host blood meals” in a specific *An. funestus* group species.

## Data Availability

The datasets used and/or analysed during the present study are available from the corresponding author upon reasonable request.

## Supplementary Materials

The following supporting information can be downloaded at: https://www.mdpi.com/article/10.3390/tropicalmed9100251/s1, Figure S1: Time series plot between annual cattle FPs of *An. funestus* species and climatic parameters in Mamfene. (A) Correlation with annual temperatures (°C). (B) Correlation with annual relative humidity (%). Year 1: 2015, Year 2: 2016, Year 3: May 2017–April 2018, Year 4: May 2018–April 2019. FPs: feeding proportions; Table S1: Mixed blood meal of *An. funestus* group species from Mamfene; Table S2: Tukey’s HSD test for multiple comparisons of mean temperatures (2015–2019); Table S3: Tukey’s HSD test for multiple comparisons of mean relative humidity (2015–2019); Table S4: Spearman’s coefficient correlation (r-values) between mean FPs of females sampled and mean monthly temperature, mean monthly relative humidity, and monthly total rainfall for archived data (January 2015–December 2016); Table S5: Spearman’s coefficient correlation (r-values) between mean FPs of females sampled and mean monthly temperature, mean monthly relative humidity, and monthly total precipitation, for newly collected data (May 2017–April 2019).
