# Supplementary material for: Host Preferences and Impact of Climate on Blood Feeding in Anopheles funestus Group from South Africa
Source: Trop Med Infect Dis. 2024 Oct 21;9(10):251. doi: 10.3390/tropicalmed9100251 (PMC11511239; doi:10.3390/tropicalmed9100251)
Supplement: Supplementary file 1 [file tropicalmed-09-00251-s001.zip › tropicalmed-3236901-supplementary.pdf]

Tropical Medicine and Infectious Disease

Supplementary Materials:

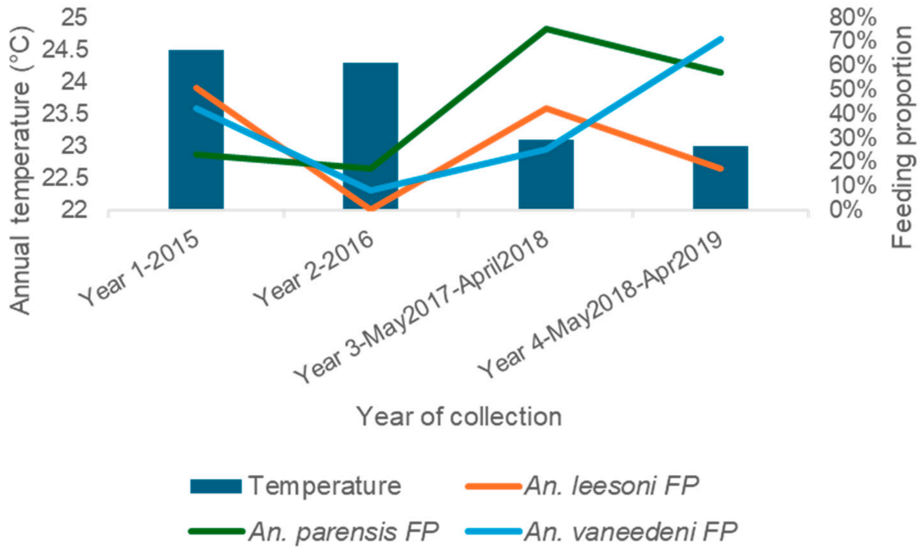

(A)

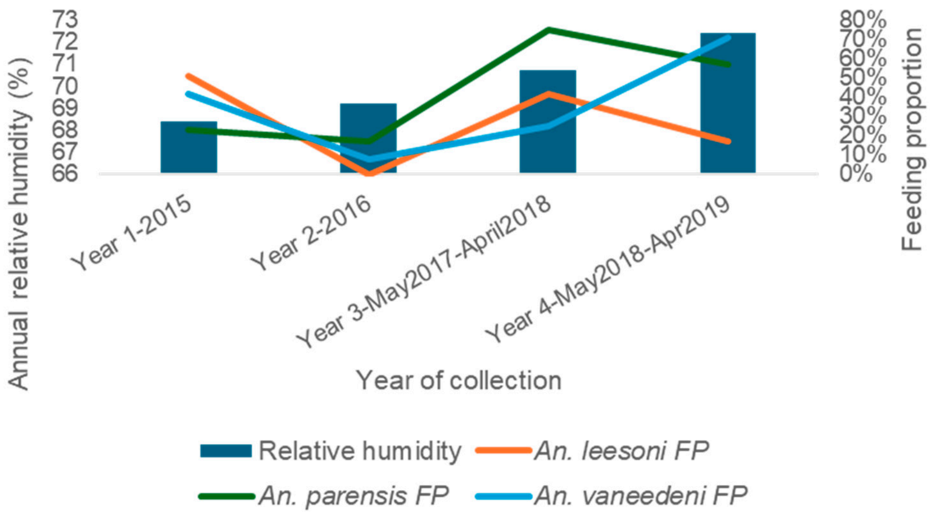

(B)

Figure S1: Time series plot between annual cattle FPs of *An. funestus* species and climatic parameters in Mamfene. A) Correlation with annual temperatures (°C). **B)** Correlation with annual relative humidity (%). Year 1: 2015, Year 2: 2016, Year 3: May 2017-April 2018, Year 4: May 2018-April 2019. FPs: feeding proportions.

Supplementary Tables

Table S1: Mixed blood meal of *An. funestus* group species from Mamfene

Species	Cattle+Goat	Cattle+Pig	Pig+Dog
An. leesoni	0	0	0
An. parensis	9	0	1
An. rivulorum	1	1	0
An. vaneedeni	0	1	0
**Total**	**10**	**2**	**1**

Table S2: Tukey's HSD test for multiple comparisons of mean temperatures (2015-2019)

Cell No.	**Tukey HSD test; variable DV_1 (Climatic parameters for 2015-2019) Approximate Probabilities for Post Hoc Tests Error: Within MS = .70125, df = 55.000**					
	**YEARS**	**2015** 24.508	**2016** 24.250	**2017** 23.367	**2018** 22.825	**2019** 23.725
2	2015					
3	2016	0.973710				
4	2017	**0.018062***	0.118736			
5	2018	**0.000239***	**0.001570***	0.612132		
6	2019	0.215250	0.643260	0.899329	0.106662	

*Marked significant differences at $p < 0.05$

The numbers in bold green represent annual mean temperatures (in °C)

Table S3. Tukey's HSD test for multiple comparisons of mean relative humidity (2015-2019)

Cell No.	**Tukey HSD test; variable DV_1 (Climatic parameters for 2015-2019) Approximate Probabilities for Post Hoc Tests Error: Within MS = 8.0016, df = 55.000**					
	**YEARS**	**2015** 68.400	**2016** 69.225	**2017** 69.583	**2018** 72.275	**2019** 70.808
2	2015					
3	2016	0.979457				

4	2017	0.907691	0.999630			
5	2018	**0.017279***	0.104657	0.199659		
6	2019	0.309993	0.743852	0.894652	0.799814	

*Marked significant differences at $p < 0.05$

The numbers in bold green represent annual mean relative humidity (in %)

Table S4: Spearman's coefficient correlation (r-values) between mean FPs of females sampled and mean monthly temperature, mean monthly relative humidity, and monthly total rainfall for archived data (January 2015 – December 2016).

**Climatic variables**	***An. leesoni***	***An. parensis***	***An. rivulorum***	***An. vaneedeni***
	r-value	r-value	r-value	r-value
Monthly temperature (°C)	-0.118	0.232	0.408	0.437
Monthly Average Relative Humidity (%)	0.120	0.593*	0.140	0.416
Monthly Precipitation (mm)	0.100	0.416	0.250	0.621*

* Correlation is significant at the 0.05 level (2-tailed), p < 0.05, 95% CI, as marked on STATISTICA

Table S5: Spearman's coefficient correlation (r-values) between mean FPs of females sampled and mean monthly temperature, mean monthly relative humidity, and monthly total precipitation, for newly collected data (May 2017 – April 2019).

**Climatic variables**	***An. leesoni***	***An. parensis***	***An. rivulorum***	***An. vaneedeni***
	r-value	r-value	r-value	r-value
Monthly temperature (°C)	-0.378	-0.252	0.538	-0.028
Monthly Average Relative Humidity (%)	-0.214	-0.088	0.655*	-0.692*
Monthly Precipitation (mm)	-0.513	-0.368	0.328	0.147

* Correlation is significant at the 0.05 level (2-tailed), p < 0.05, 95%, as marked on STATISTICA
